# New products

**DOI:** 10.1155/S1463924685000487

**Published:** 1985


Journal of Automatic Chemistry/*Journal of Clinical Laboratory Automation*, Vol. 7, No. 4 (October–December 1985), pp. 209–217

### New products

### Shaking water-bath

The Shaking Water Bath from Tecator provides accurate temperature control and flexible mixing of liquids and suspensions. A series of exchangeable flask trays offer high incubation capacity for a wide range of sample sizes. The electronically controlled thermostatic water-bath has a mechanically driven shaking trolley. A transparent lid ensures temperature stability within ±0.1 °C up to 80 °C and prevents evaporation loss. Temperature control is done either automatically by presetting of up to three optional temperatures, or manually. The water temperature is digitally displayed and a cut-out prevents overheating. The shaking speed is continuously adjustable within the range 10–150 strokes/min and the stroke length can be varied up to 50 mm.

*More information from Tecator AB, Box 70, 5 26301 Höganäs, Sweden. Tel.: 042 423 30.*

**Circle No. 99 on Reader Enquiry Card**

### Air sampling

A range of portable air samplers, manufactured in Italy, has been introduced into the UK by SKC Ltd. The 'LWS' samplers contain a diaphragm pump providing high constant flow facilities to minimize the effects of filter build-up in dusty environments. A liquid crystal display illustrates sample volume, sample time and temperature. There are built-in audible and visual alarms, which warn of electricity supply faults or malfunctions, and two independently adjustable flowmeters cover the range of .1 to 25 l/min. Also incorporated is a delayed start feature, allowing extended operational flexibility; a sampler time programmer is available for selection of sample periods between 1 and 9999 min.

*Full details from SKC Ltd, Hamworthy Trading Estate, Dawkins Road, Poole, Dorset BH15 4JW, UK. Tel.: 0202 671121.*

**Circle No. 100 on Reader Enquiry Card**

### Satellite GC

Packard Instrument offer a fourth model of gas chromatograph, which can be used in their 'Network' system. The Model 436S satellite gas chromatograph features the same capillary performance as Packard's Model 436. It is identical in every way, but has no programming facilities and thus offers considerable cost savings over conventional GCs.

The oven has temperature gradients as low as ±0.1 °C. Other features include high-speed electrometers, proportional oven temperature control, quality insulation, microprocessor control, non-turbulent column environment, and complete integral flow control units optimized for the latest column technology. The Model 436S is ready to accept microbore columns without modifications.

*Details from Packard Instrument Ltd, 13–17 Church Road, Caversham, Berkshire RG4 7AA, UK. Tel.: 0734 478234.*

**Circle No. 101 on Reader Enquiry Card**

### Monarch

The new Monarch Chemistry System from Instrumentation Laboratory is described as a consolidated, easy-to-operate and cost-effective system for analysing electrolytes, routine chemistries, special proteins, and therapeutic drugs, thus meeting up to 98% of current laboratory requirements. The system utilizes multiple detection techniques, including absorbance, fluorescence, light scatter, and potentiometry. And the Monarch offers the user a choice of test modes: random access patient-prioritized, time-optimized, and stat sampling.

The instrument couples an on-board capability of 23 tests (using ISE) with fast, easy change-over to additional IL-developed tests and verified applications for EIAs, FIAs and special chemistries. Programmable capacity on the Monarch system runs as high as 100 tests.

For convenience, IL-Test reagents come in prefilled BoatIL containers which can be transferred directly from the package to the system's reagent tray. The BoatIL containers include bar-coded labels, which the system scans prior to analysis to assure that the requested reagents are loaded on the tray; the bar-coding feature also allows operators to load multiple containers of the same reagent on one reagent tray.

Test set-up consists of a few basic keystrokes on the system's modifiable direct action keyboard. Users can program and identify—on a single key—the profile groups and individual tests most often requested in the laboratory.

Once testing starts, the system's software co-ordinates micro-parallel analysis and robotic cuvette transport—attaining throughput of up to 600 tests. The Monarch also performs the following functions, each in parallel: loading and sample analysis; chemistry and electrolyte analysis; and analysis on all cuvettes.

*Further information from Instrumentation Laboratory, 113 Hartwell Avenue, Lexington, Massachusetts 02173, USA. Tel.: 617 861 0710.*

**Circle No. 102 on Reader Enquiry Card**

### Potentiometry

Radiometer of Copenhagen have for many years been the leaders in potentiometry technology; four new clinical applications have recently been prepared using Radiometer analytical instrumentation. The first is the determination of fluoride in tooth enamel using the Radiometer Ion 85 or Ion 83 specific analysers. Secondly, the applications laboratory have prepared information on the titration of gastric aspirate using the ETS 822 system and also pancreatic function testing. Finally, a further titration application for free fatty acids in sebum useful in dermatological studies.

The Ion 85 or Ion 83 specific-ion monitoring system can be used in conjunction with a fluoride-ion selective electrode to assist in the evaluation of dosing drinking-water with fluorides, as well as the rate of uptake of fluoride by tooth enamel.

Gastric aspirate titrations are used to monitor acid production in the stomach, the samples tending to be both viscous and discoloured, thus making manual titrations difficult. With a Radiometer ETS 822 end-print titration system this task can be fully automated giving results on just 2 ml of aspitate.

The other clinical application using the Radiometer ETS822 is the pH stat titration for trypsin activity (a pancreatic function test). Using the ETS in pH stat mode with just 100µl of gastric aspirate, accurate trypsin activities can readily be determined.

Finally, the Radiometer MTS 800 System can be used to titrate free fatty acids present in skin swabs. This method is currently being used to evaluate the effects of acne creams and in other dermatological surveys.

*Details from V. A. Howe & Co. Ltd, 12–14 St Ann's Crescent, London SW18 2LS.*

**Circle No. 103 on Reader Enquiry Card**

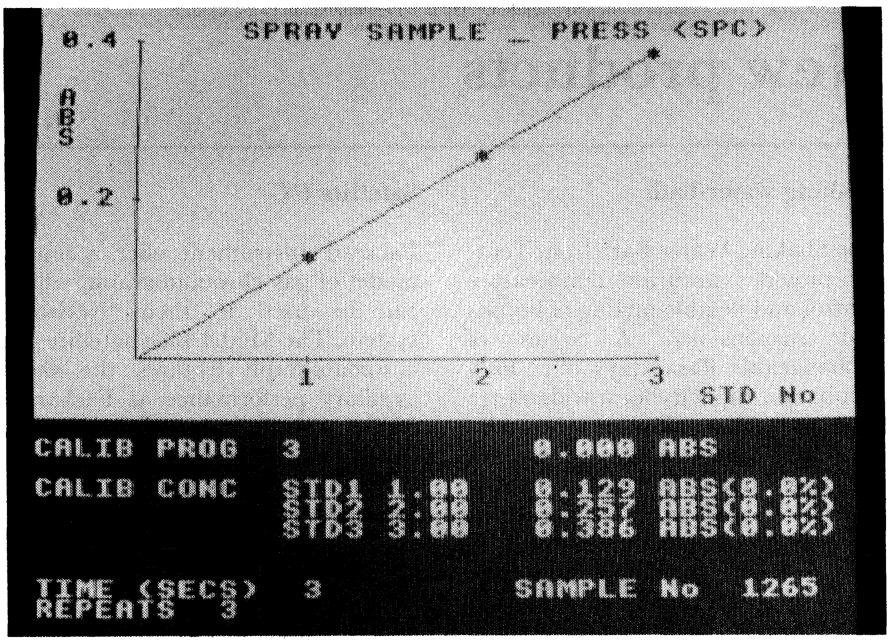

*A new data system for the Philips Analytical SP9 atomic absorption range offers extensive facilities, including full colour graphics, at a saving of many thousands of pounds compared with a dedicated system. Based on the BBC micro, the package features software written by a Philips Analytical AA expert, which gives all the capabilities required of a flame graphics system. These include cookbook information, live absorbance display, data recall with weight correction, calibration display, calibration with up to five standards, storage of calibration curves, statistical information and a formatted report with print-out of the calibration display. As the system is menu-driven, only nominal operator skills are required to obtain the full benefits of the software. Contact Pye Unicam Ltd, York Street, Cambridge CB1 2PX, UK.*

**Circle No. 105 on Reader Enquiry Card**

### HPLC gradient controller

The Model 1025 gradient controller is intended for binary elution gradients in HPLC. Up to 25 segments of time, %B and flow can be used to fully define a profile from a simple linear gradient to a complex curved or stepped gradient. Within each profile, the user can nominate as many as 10 independent relay events to trigger external devices during the run. A total of 10 gradient profiles can be stored in RAM for immediate implementation. The RAM is protected against any forms of mains failure by integral battery back-up.

The compact Model 1025 will form gradients in both high-pressure and low-pressure mixing systems. A low-pressure mixing module (Model 1020) is available as an accessory, working equally effectively with manual and automatic sample injection methods. Any matrix of the 10 profiles can be nominated for up to 100 samples, and with automatic sample loading, the controller can operate completely unattended.

This new Drew instrument is reported as having many design features to provide optimum convenience and the flexibility essential for analytical methods development. For example, peaks can be opened out for close examination or flushed out for lack of interest by single keystrokes. Real Time Profile Editing (RTPE) allows the user to change a gradient profile and the auxiliary relay events without interrupting a run. A small printer is also built into the Model 1025 enabling the user to check a hard copy of the profiles before storing in RAM and starting analysis—thus saving valuable time which might otherwise be lost through false starts and aborted runs.

The instrument is compatible with a variety of pumps, autoloaders, fraction collectors and integration systems.

*Further details from Drew Scientific Ltd, 12 Barley Mow Passage, Chiswick, London W4 4PH. Tel.: 01 995 9382.*

**Circle No. 104 on Reader Enquiry Card**

### DEM cells

Energy-saving technology developed at the Electricity Council's Capenhurst Research Centre has begun to prove itself at a chemical manufacturing plant in Surrey. Dished electrode membrane (DEM) electrochemical cells installed by Steetley Chemicals Ltd at its works near Cranleigh have already demonstrated that they are cleaner and both easier and cheaper to run than the company's present bromate production plant. Capenhurst hopes that operation of the plant will herald widespread adoption of the technology by industry. DEM cells can be used in the manufacture of pharmaceuticals, dyestuffs and cosmetics as well as inorganic compounds, and they are also useful for recovering metal and other chemicals from solution.

Steetley Chemicals Ltd is a subsidiary company of Steetley PLC; another subsidiary, Streetley Engineering, was licensed by the Electricity Council in 1983 to manufacture and market the cell. It was developed to permit the use of higher current densities than in traditional parallel plate cells, thereby achieving improved electrical efficiency.

The DEM cell has obvious advantages over Steetley Chemicals' present equipment. Energy costs are between a quarter and a third lower. A single DEM unit does the work of 12 of the existing cells – yet occupies no more space than one of them. In addition to saving production space, a plant composed of DEM cells has fewer electrical connections. Because it is an enclosed system, there is less risk of spillage and product contamination is avoided. It is also safer because solids build-up is avoided in the electrode area and quality checks can be made by taking one sample from a single cell instead of one from each. Finally, faster switching from the manufacture of one grade of product to another enables Steetley Chemicals to match its output more closely to the demands of the market.

The DEM cell has been shown to be able to synthesize a range of both organic and inorganic chemicals including naphthaquinone, aromatic aldehydes, aliphatic and aromatic carboxylic acids, chlorates and hypochlorite. Proven materials-recovery processes include regeneration of chromic acid and other oxidants and the recovery of caustic soda and acid from spent sulphates.

A further advantage of the cell is that it is relatively easy to scale up from a pilot plant to full production. The dished electrode design with small inter-electrode gaps of between 2 and 7mm gives excellent flow characteristics enabling high productivity rates to be achieved. Three sizes of cell are available with single electrode areas of: 0.05, 0.175 and 1.0m$^2$. All three may be extended to accommodate up to 50 electrode pairs.

*Further information about the DEM cell is available from Steetley Engineering Ltd, PO Box 20, Lenches Bridge, Kingswinford, West Midlands DY6 8XA, UK. Tel.: 0384 55941.*

**Circle No. 106 on Reader Enquiry Card**

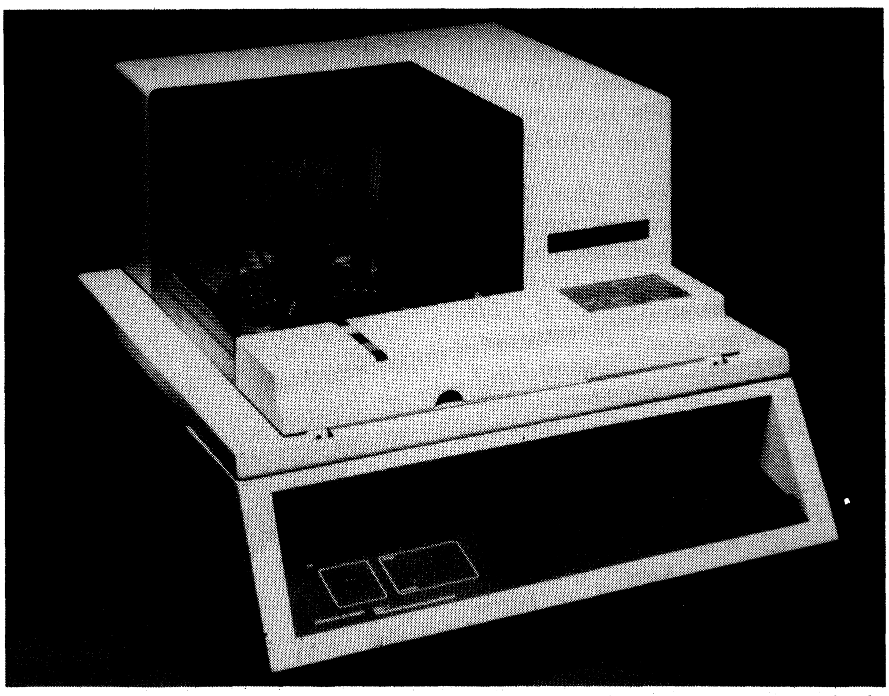

*Perkin-Elmer have expanded their TADS Series thermal analysis system with the introduction of a new DSC-4 Robotic System, which is compatible with new and existing DSC-4 differential scanning calorimeters. Using this robotic system, it is possible to run 48 DSC samples without operator intervention. The DSC-4 comprises a removable, 48-position sample carousel and a pneumatically controlled robotic sampling arm. The robotic arm automatically selects a desired sample from any of the 48 carousel positions, places it in the DSC-4 sample holder, and closes the sample holder enclosure cover. The DSC experiment, which has been set up in the Robotic System software program on the thermal analysis data station (TADS), is then automatically performed. When the experiment is complete, the data is automatically stored on disk and, if necessary, a repeat experiment is performed on the same sample. When a run is complete, the robotic arm removes the sample, replaces it in its original position on the carousel, and selects another sample for analysis. This sequential or non-sequential sample selection allows up to 48 samples to be analysed in any order chosen by the user. For further information contact Perkin-Elmer Ltd, Post Office Lane, Beaconsfield, Buckinghamshire HP9 1QA, UK. Tel.: 04946 6161.*

**Circle No. 107 on Reader Enquiry Card**

### Epsilon hCG

Beckman Instruments, Inc. has introduced the Epsilon Enzyme Immunoassay High Performance 'β Specific' hCG (human chorionic gonadotropin) test for serum or urine samples. The test offers a 15 + 15 min qualitative protocol, a 20 + 20 min quantitative protocol and an optional high-sensitivity procedure. Single-point calibration saves up to six tubes per run yet maintains accuracy. The test is easy to perform with one-step antibody incubation.

Advanced proprietary bead coating technology applies an even layer of monoclonal antibody on all beads simultaneously for a consistent, reproducible assay. Alpha- and beta-specific antibody pairs measure intact βhCG with no interference from α and β subunits. The test is usable for diagnosis of ectopic pregnancy.

The Epsilon EIA test measures concentrations in two ranges – 2 to 400 mIU/mL and 0.5 to 100 mIU/mL. Results correlate with RIA – ($r$) = 0.98 – and protocols are short and easy to learn.

The assay can be processed on the 60-tube Epsilon Test Processor to capture beads for a quick, secure wash. Results are read on any spectrophotometer at 492 nm. It exhibits minimal cross reactivity with pituitary hormones or related compounds and requires sample volume of only 50 μL. It is standardized against the WHO first IRP (1975).

Each kit contains reagents and standard for 100 tests. Reagents are stabilized for long shelf life at refrigeration temperatures with minimum lot to lot variation. Other test assays in the Epsilon Immunoassay line are IgE, TSH and Digoxin.

*Beckman's international offices are as follows: USA — Beckman Instruments, Inc., 2500 Harbor Boulevard, Box 3100, Fullerton, California 92634; Australia — Beckman Instruments Australia Pty. Ltd, 24 College Street, Gladesville, New South Wales 2111; Japan — Beckman Instruments (Japan) Ltd, 2nd Floor, Daiichi-Nano Bldg., 2-21-2, Nishi Shinbashi, Minato-Ku, Tokyo; Other Asia — Beckman Instruments (Hong Kong) Ltd, 15th Floor, Unit B/D, Gee Chang Hong Centre, 65 Wong Chuk Hang Road, Aberdeen, Hong Kong; Other territories — Beckman Instruments International S.A., P.O. Box 308, 1211 Geneva 6, Switzerland.*

**Circle No. 108 on Reader Enquiry Card**

### Information displays and automation

To simplify the designer's job of interfacing displays with automated systems, Displait Ltd now offer STAR. This is the acronym for Serial Transmitter And ROM, and is a combined message store and transmission unit. In conjunction with Displait's Locaterlarm series of alphanumeric read-outs, it is designed to tell machine supervisors what their plant is doing. Complete with mains-power supply and requiring only a basic BCD output from a programmable controller, it leaves engineers free to get on with designing their system. The primary application is for fault indication: a fault message, clearly spelled out, is quickly acted upon. A greater range of fault situations can be presented, no operator training in code translation is required and code books become a thing of the past. STAR is vital where supervisors cannot see all the machines under their care. As an information display, this system has already been used in the food processing industry on multi-product process lines to indicate the product currently on line.

*Technical description from Displait Ltd, 66 Londesborough Road, Scarborough, North Yorkshire YO12 5AJ, UK. Tel.: 0723 352103.*

**Circle No. 109 on Reader Enquiry Card**

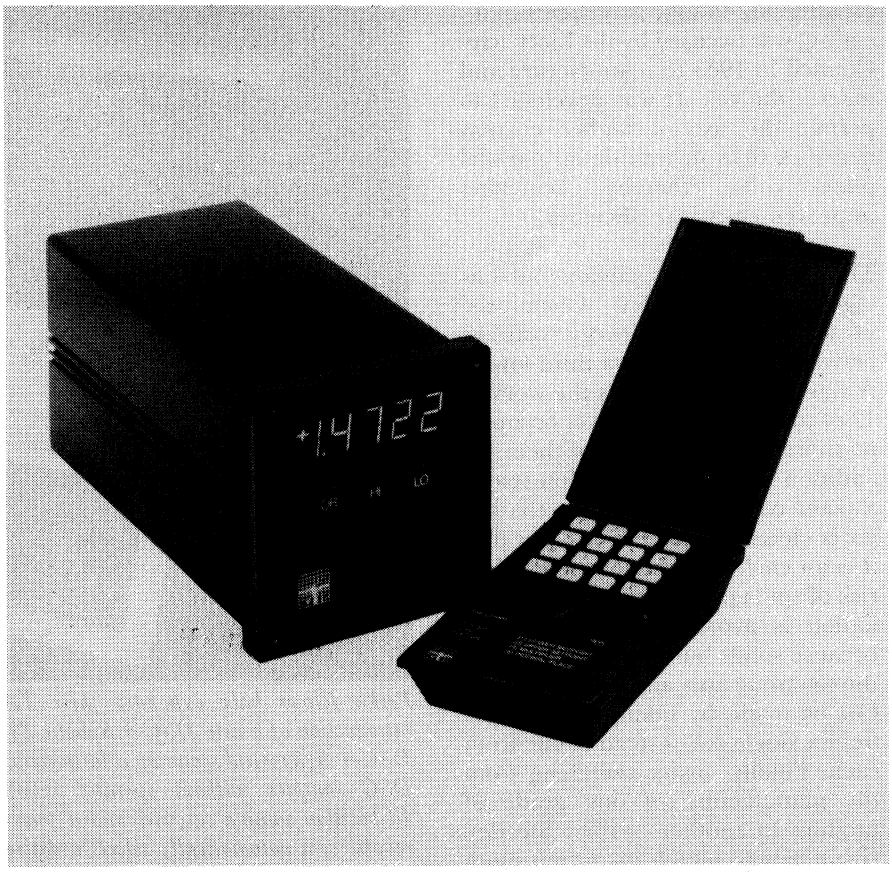

*The YSI 4500 Series Data Grabbers, which provides integrated data acquisition and control for industrial processes, laboratories and other multiple-sensor applications. This instrument conditions, digitizes, linearizes, displays and transmits signals from up to 128 sensors; it also reports alarm conditions. It can stand alone with its detachable keyboard, or it may be interfaced with a computer for two-way communications. A 4½-digit LED display, with selectable decimal point, provides read-out of sensors and alarm conditions. Resolution of bipolar signals may be as high as 0.025%, and signals may be linearized piecewise with 512 segments. The aluminium DIN standard case provides RF shielding. Input signals may be bipolar current, votage, time-based, resistance or frequency. Details from YSI at Yellow Springs, Ohio 45387, USA. Tel.: 513 767 7241.*

**Circle No. 110 on Reader Enquiry Card**

### HILDA: HPLC controller and gradient programmer

An inexpensive, flexible automation of test runs is possible with this user-programmable process-controlled system. It operates on a real-time digital time base principal (user settable): switching of six solvent valves, control of two gradient makers (low pressure), or 4, 0–10V output ramps to control suitable pumps for high-pressure gradient making, two closing contacts for operation of auto-injectors, or similar and eight mains-operated devices such as pumps, chart recorders, heaters etc., up to 300W each (total 1.5 kW max) is provided. Gradients and whole programs can be stored and recalled at will.

HILDA is mains-failure protected and will operate a pre-set mains-failure recovery program when power returns so that time is not wasted. The user is presented with a hard copy of gradients generated and a print-out of all timings.

LabLogic believe that HILDA will find many applications, particularly with those workers who are doing methodology development, or have to run different samples under varying conditions.

*Details from LabLogic Ltd at Petre House, Petre Street, Sheffield S4 8LJ, UK. Tel.: 0742 432933.*

**Circle No. 111 on Reader Enquiry Card**

### GPC

Gel permeation chromatography (GPC) is a mode of liquid chromatography in which sample molecules are separated according to molecular size. The technique is widely used in the organic polymer industry, where it can provide rapid evaluation of the composition of raw materials and final products.

Perkin-Elmer's new GPC systems demonstrate the importance of a co-ordinated system approach in realizing the full benefits of high-resolution, high-speed GPC for quality-control applications. A typical system is designed around the Series 10 isocratic pump, which is suited to dedicated GPC applications because of its ease of use and flow rate stability. The two most common types of detector used in GPC – refractive index and ultra-violet absorption – are available for the system. The LC-25 refractive index and LC-95 UV/Vis detectors provide low instrumental band broadening, low noise and fast detector response.

'Mixed bed' GPC columns, with a broad operating range of over four decades in molecular weight, are included. These columns reduce analysis times to minutes, rather than hours, with a corresponding saving in solvent consumption.

Raw data are acquired and stored on disk using the Chromatographics 2 chromatography data system based on the Model 3600 Data Station. GPC5 data-processing software can then be used to interpret the stored data. The software is extremely easy to use through the keyboard and screen of the Model 3600. It computes column calibration curves, molecular weight averages and normalized molecular weight distribution. The results of these calculations may also be stored on disk. Molecular weight averages calculated from GPC data can be produced to within less than 1%.

*For further information contact Perkin-Elmer Ltd, Post Office Lane, Beaconsfield, Buckinghamshire HP9 1QA, UK. Tel.: 04946 6161.*

**Circle No. 112 on Reader Enquiry Card**

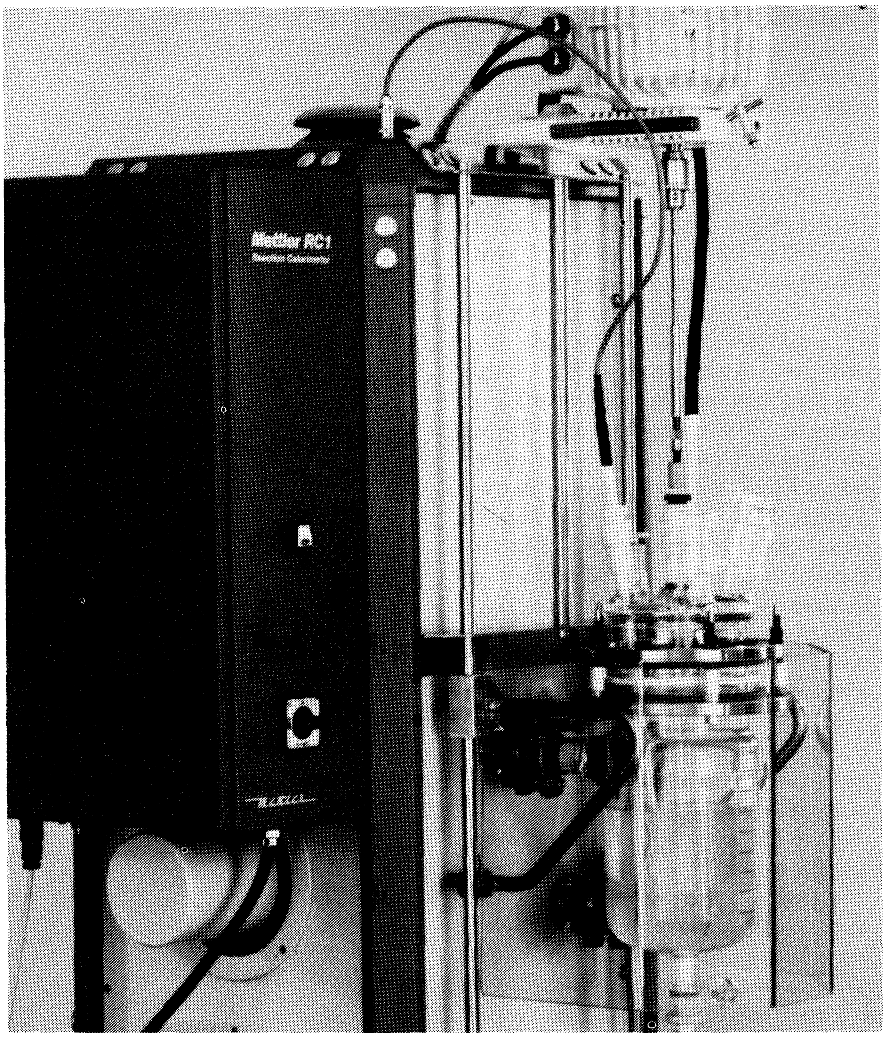

*The RC1 reaction calorimeter from Mettler which measures heat flow in chemical reactions and physical transformations. All reaction design data can be determined automatically and reproducibly under conditions close to those during actual operation. The RC1 consists of a twin-walled reactor (effective volume 2 l) and a glass cover with several openings for measuring sensors and dosage additions. A heat transfer medium flows between the inner and outer walls of the reactor to allow heat to be supplied to or withdrawn from the reaction medium. A stirrer, a thermostat and an electronic control unit are also fitted. The control unit carries out the instructions entered by the user on the attached PC. The RC1 is suitable for use in process development or for safety investigations and trial production runs. Full information from Mettler Instrumente AG, CH 8606 Greifensee, Switzerland.*

**Circle No. 113 on Reader Enquiry Card**

### VEGA – a new GC

Erba Science, the UK subsidiary of Carlo Erba, has introduced a compact gas chromatograph for all column types. The principal feature of the VEGA is its ability to accept a wide range of detectors and injectors, all controlled from the keyboard, in a unit that will take up 19 in of a bench. Included in the range of injectors is the Carlo Erba cold on-column injector. User-friendly control is achieved through an interactive keyboard/video and a multi-ramp temperature programmer. Two RS 232C output ports are included as standard. The instrument is also fully modular with existing Carlo Erba accessories; this makes it suitable for individual or automatic analysis with cold on-column, headspace, cryogenic focusing or liquid injectors.

Instrument programming and control functions are all entered through the keyboard. Comprehensive menus are provided on the video display, where the required parameters are entered with the aid of a cursor. Two programs may be entered and retained for recall as required: for example, they may be activated in turn during a series of automatic analyses in conjunction with an autosampler. A novel feature of the VEGA's temperature programmer is that the alternative program may be recalled to the CRT and edited while the other program is operating, without in any way affecting the storage or implementation of the operating program. Alternatively, the operating program may be edited during an analysis. The video will also display all program parameters in conjunction with a real-time display of oven temperature progress through a multi-ramp program. While events control (column switching, back flushing etc.) are all pre-programmable, this also allows the operator to initiate such functions at will.

Included in the VEGA is the Carlo Erba proportional cooling system for near-ambient operations: control of the system as required is automatic at program end, or by manual override from the keyboard. Other standard features of the control program are automatic control for split/splitless or on-column injectors and a comprehensive self-diagnosis program. In the latter case displayed codes are referred to a list of possible faults in the instrument manual. The controller also includes a trickle-charged back-up battery capable of maintaining stored programs for four days during mains disconnection or failure.

*Full technical information on the VEGA gas chromatograph is available from Erba Science (UK) Ltd, Headlands Trading Estate, Swindon, Wiltshire SN2 6JQ, UK. Tel.: 0793 33551.*

**Circle No. 114 on Reader Enquiry Card**

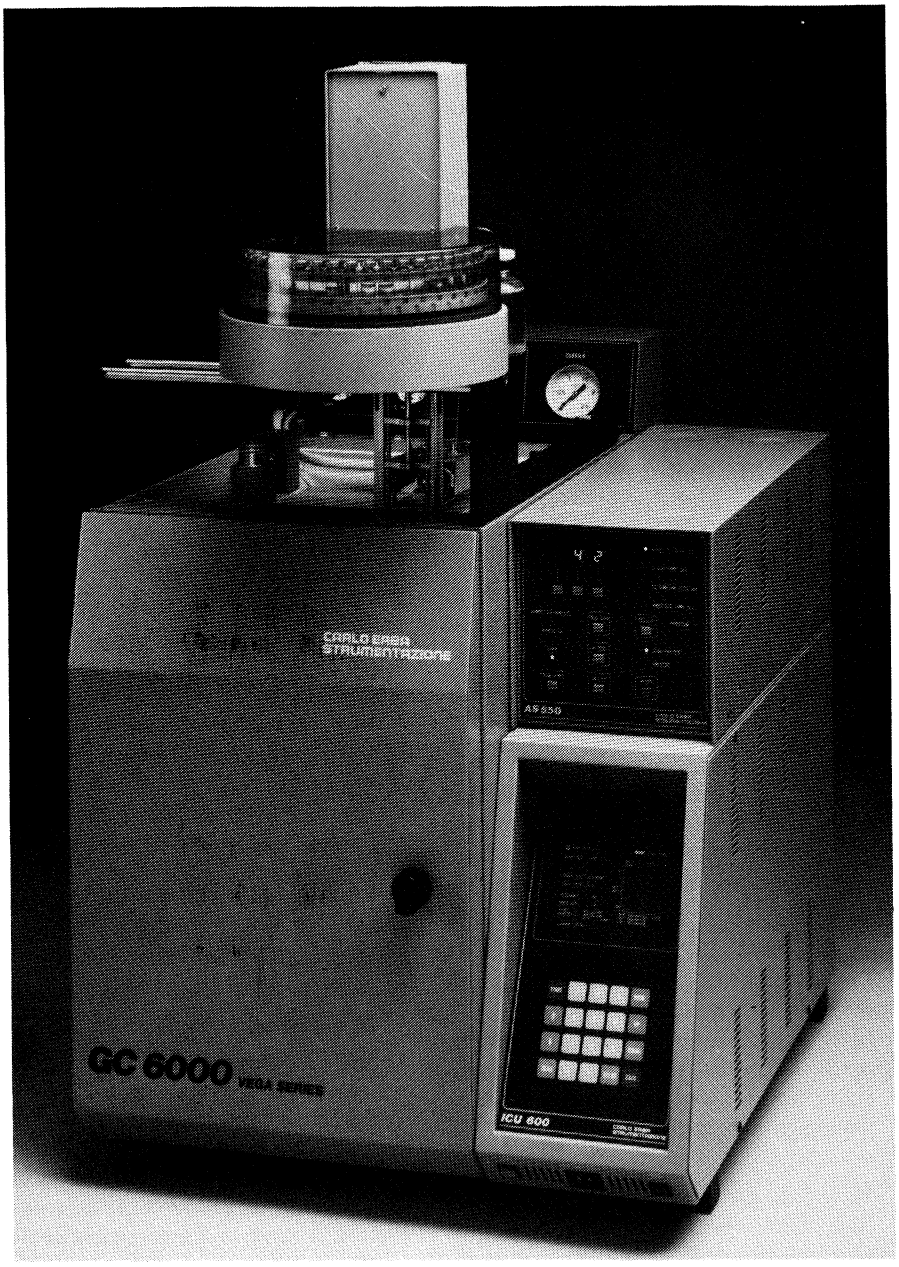

*Carlo Erba feel that the VEGA will be an ideal instrument for the QC lab and other routine applications, where its sophisticated performance, ease of use, small physical size and price make it an attractive working tool. Its compatibility with existing autosamplers including the cold on-column autosampler, and other accessories, however, should also make it attractive to the existing research laboratory wishing to extend its GC capability at moderate cost.*

### Corning Glass & Ciba-Geigy

Ciba-Geigy Ltd and Corning Glass Works have announced that they have agreed to form a new company, Ciba Corning Diagnostics Corporation, to pursue the $5 billion worldwide market for diagnostic products.

The 50–50 owned company has been approved in principle by the boards of both organizations. Completion of the transaction is subject to the execution of definitive agreements and certain governmental approvals.

Corning will contribute its present laboratory diagnostic products business to the new firm. This business, with annual sales of about $160 million, markets its products under the Corning Medical and Gilford names. Ciba-Geigy will provide access to its life-science research capability.

Officials said the formation of Ciba Corning Diagnostics meets the strategic objectives of both parents. Ciba-Geigy, a major pharmaceutical company, has decided to participate in diagnostics. Corning's present diagnostics business has been searching for an association with a world-class life science research centre.

*Details from Corning Medical and Scientific, Corning Ltd, Halstead, Essex CO9 2DX, UK. Tel.: 0787 472461.*

**Circle No. 115 on Reader Enquiry Card**

### T.A.M.E.D. (Totally Automated Methods Development)

T.A.M.E.D. is a software suite that is run on the LDC/Milton Roy Chromatograph Control Module (CCM) to eliminate the time-consuming trial-and-error process of HPLC methods development. The software enables the CCM to automatically determine the optimum flow rate and solvent concentrations, in order to obtain the best possible chromatographic resolution for any given sample.

Operator intervention is not required with the system, so automated methods development can be performed overnight. Although the system is fully automated, manual override is always available so the operator never loses control. No prior knowledge of the sample is required, i.e. sample type, or number of components present as the T.A.M.E.D. software seeks the maximum number of components with optical chromatographic resolution. However, the software allows known information to be input, thereby speeding up the optimization procedure.

T.A.M.E.D. enables both binary and ternary solvent mixtures in the isocratic mode, and binary multistep gradient separations, to be optimized. The chromatographer is not limited to reverse phase chromatography, normal phase and ion pair separations can also be optimized.

*More information from Laboratory Data Control (UK) Ltd, Milton Roy House, High Street, Stone, Staffordshire ST15 8AR, UK. Tel.: 0785 813542.*

**Circle No. 116 on Reader Enquiry Card**

### Distillation

The addition of a new distillation unit to their range of Nitrofoss Semi-Automatic Kjeldahl equipment is announced by Foss Electric (UK) Ltd. Water and sodium hydroxide are added by means of a push-button on the front panel, reducing the chemical handling normally associated with manual Kjeldahls. The unit also incorporates an automatic timer to enable distillations to switch off automatically. This is particularly useful for the determination of sulphur dioxide, where fixed time distillation is required.

The Nitrofoss system offers four- and eight-place digestion blocks, which may be run at half capacity, and incorporates an efficient fume extraction system which removes all acid fumes through a simple water jet vacuum pump. This means that there is no requirement for a fume cupboard. Because the heat is by infra-red radiant heaters, the temperature of digestion is achieved without the use of hydrogen peroxide and complete digestion can be achieved in under 30 min. When combined with the new distillation unit, full Kjeldahl analyses may be carried out in under 1 h. After distillation, the hot alkaline solution is automatically removed to drain.

*For further information contact Graham Lee at Foss Electric (UK) Ltd, The Chantry, Bishopthorpe, York YO2 1QF, UK. Tel.: 0904 707944.*

**Circle No. 117 on Reader Enquiry Card**

### P.S. Analytical expands range of ICP and atomic absorption accessories

To complement its Hydride Generator System PSA 10.002 and the associated autosampler, P.S. Analytical has launched a new range of ICP and AA accessories. Foremost amongst these are the Ebdon High Solids Inert Nebulizer, the Four-Channel Diluter Peristaltic Pump and the Drain Bucket Level Alarm Sensor. Each increases the efficiency of the ICP/AA operation and allows the user to make the most of his expensive instrumentation.

The High Solids Nebulizer was developed by Dr Les Ebdon's group at Sheffield Polytechnic and then at Plymouth Polytechnic. Originally designed for slurry nebulization, its sphere of operation has been further extended by choice of construction material and physical dimensions so that it offers a viable alternative to conventional cross-flow nebulizers and has the additional advantage of being made from inert materials. It can therefore be used with strong acid solutions and particularly with HF solutions.

The Four-Channel Peristaltic Pump has been proven as a reliable introductory system into ICP and AA, particularly when used with the above nebulizer. In addition, it has extremely reproducible performance as a diluter pump and CVs of better than 0·2 has been obtained from repeat analysis of sodium and potassium levels by flame photometry using a 1:200 dilution. The pump operates at 1.500 r.p.m. and a wide range of flow-rates are available.

The Level Alarm Sensor has been designed to provide a simple and effective means of preventing spillages on drain vessels. The device also has additional contacts available so that instruments can be automatically shut down; alternatively, an external pump can be activated to drain the waste vessel through the normal mains drainage until a safe state is achieved.

PS Analytical also markets plasma torches, spray chambers and nebulizers for ICP and DCP applications.

*For further details contact P.S. Analytical at Arthur House, Far North Building, Cray Avenue, Orpington, Kent BR5 3TR, UK. Tel.: 0689 31632/3.*

**Circle No. 118 on Reader Enquiry Card**

### Spectroradiometers

Spectron Engineering has introduced RapidScan Very High Speed (VHS) Spectral Scanning options for their SE490 line of visible, UV, and near-IR spectroradiometers.

The most dramatic RapidScan VHS feature records time-resolved spectra, each consisting of 256 adjacent spectral bands, at an operator controllable rate up to a 2000 Hz.

High-speed acquisition of continuous spectra has research, product development and testing, and process-control applications. It is suited for analysis of dynamic events or cycles where the spectral emission or absorption may be changing rapidly.

The RapidScan VHS Spectral Scanning could characterize spectral variations, such as phosphor decay curves, within the operating cycle of fluorescent lamps (120 Hz frequency) or other light sources, plasma dynamics, moderate speed flash events, or even some explosions. The system could be used for high-speed spectroscopy. Spectral absorption of rapidly changing process variables could be monitored using a flow cell and a synchronized strobe light source.

The SE490 line uses a photodiode array detector to measure a continuous spectrum simultaneously in 256 adjacent spectral bands. The spectral dispersion is 1·6 nanometers per channel for the 370–730 nm visible detector, 0.9 nm for the 200–400 nm UV detector, and 2.9 nm for the 390–1100 nm wideband detector. The spectroradiometers include a microprocessor-based controller which can display, print or output (RS-232) spectral data under operator control.

A second RapidScan operating mode is included with the VHS feature and also offered separately. It uses the standard scan rates, for example a 30 Hz rate, and it allows the user to define the number of spectral scans desired and an interval (0 to 99 s) between scans. Up to 96 spectra, each stored as 256 channels with 12 bit resolution, are automatically retained in memory. RapidScan VHS stores 192 (8 bit) spectra in memory.

Both the RapidScan and RapidScan VHS features give complete flexibility in reviewing stored spectra. Direct random access is provided to individual or pairs of spectra which can be displayed on the built in CRT or printed for a hard-copy record. Any individual spectrum or the entire set of spectra in memory can also be transmitted via an RS-232 port to the user's computer.

*For more information, contact Spectron Engineering, Inc., 800 W. 9th Ave., Denver, Colorado 80204, USA. Tel.: 303 623 8987.*

**Circle No. 119 on Reader Enquiry Card**

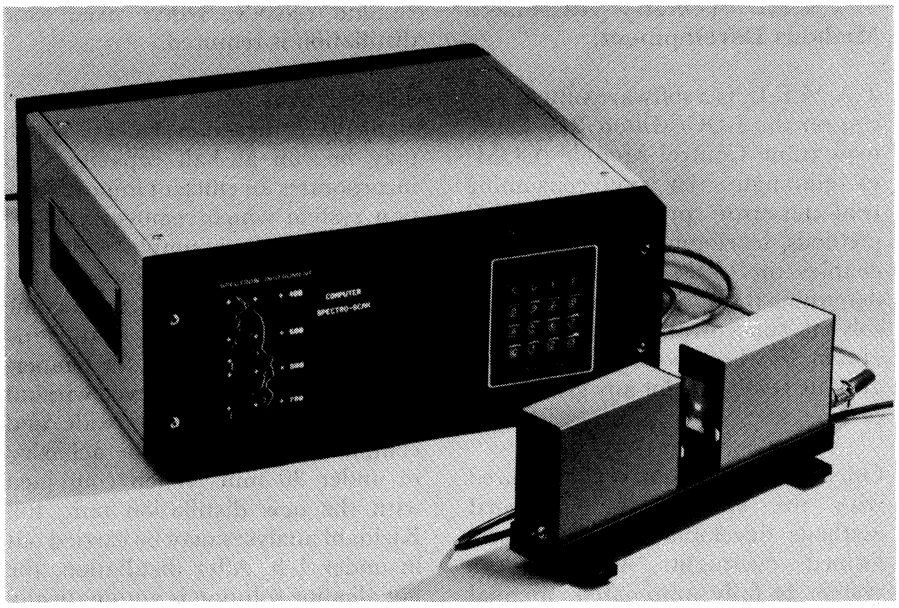

*The CE490 Spectroradiometer which is now available with the RapidScan Very High Speed Spectral Scanning option which takes up to 2000 continuous high resolution spectra/second. (Spectron Engineering, Inc., Denver, USA.)*

### Industrial Process Computer

An industrial process computer has been added to the range of equipment from Paar Scientific. The ITB IZO is suitable for use in process and quality-control applications and is expected to find wide acceptance throughout industry – including breweries and dairies and in particular food, soft drinks, paint, petrochemical, pharmaceutical and chemical industries.

The ITB 120 operates using instructions which have been externally keyed in via the touch-sensitive alphanumeric keyboard. It can also receive data from a variety of instruments including pH meters, viscometers, flow or level meters etc., and its modular design permits up to 128 inputs to be accommodated.

This instrument also features digital display, 16K Eprom extendable to 32K, 8K RAM extendable to 16K non-volatile memory and 2K RAM extendable to 8K on the computer, two each density, temperature and analogue inputs together with six logic inputs as standard, and two analogue, eight logic and two RS 232 outputs on the basic version.

The ITB120 is available as a bench model, for rack, wall or partition mounting or sealed in a watertight container.

*Details from Paar Scientific Ltd, 594 Kingston Road, Raynes Park, London SW20 8DN. Tel.: 01 542 9474.*

**Circle No. 120 on Reader Enquiry Card**

### Sodium electrode

A new ion-selective electrode from Fisher is designed for sodium measurements wherever sensitive discrimination between sodium levels is required. This includes not only biological samples, water treatment, seawater intrusion studies and general process control, but food technology, where the sodium content of foodstuffs is of increasing concern.

The electrode permits direct potentiometric measurement of sodium ion activity in aqueous solutions. It exhibits excellent Nernstian response from 1 to $10^{-6}$ molar sodium concentration, and will give analytically useful results down to $10^{-7}$ molar, in the 0° to 80 °C range.

The body of the Fisher unit is rugged polymer, making it especially suitable for demanding applications in food technology.

Another feature: although the new model is, for maximum convenience, a combination electrode, it has a calomel instead of a silver chloride reference. This minimizes junction clogging, which occurs when a sample's protein reacts with silver.

*Details from Fisher Scientific, 711 Forbes Avenue, Pittsburgh, Pennsylvania 15219, USA. Tel.: 412 562 8468.*

**Circle No. 121 on Reader Enquiry Card**

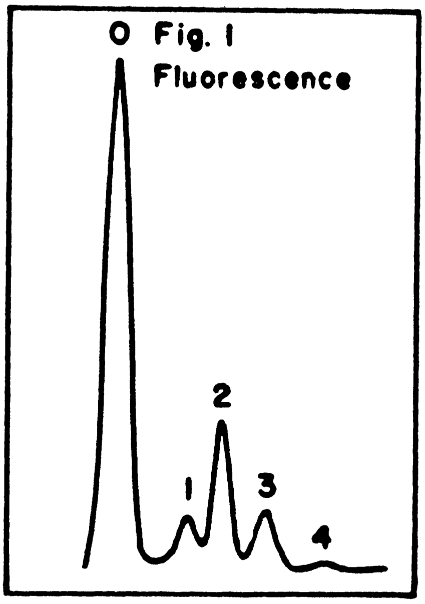

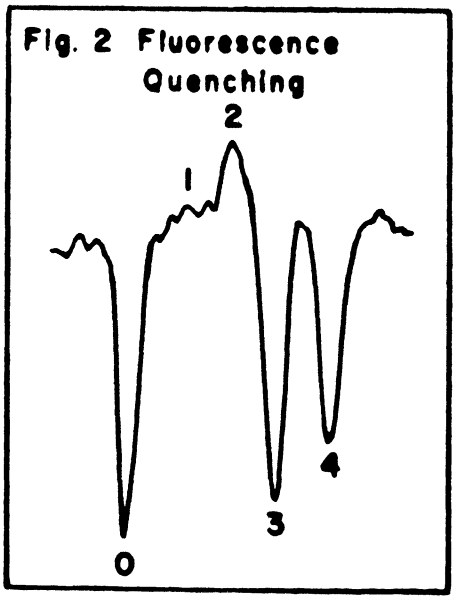

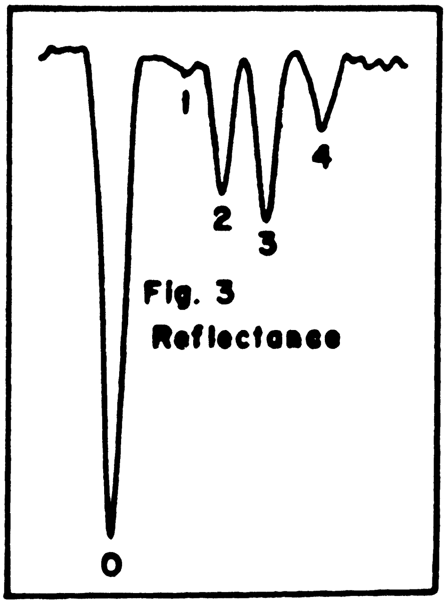

*The S.I./McPherson Model FLA-750-006-0 TLC Plate/Gel Scanner adapts the S.I. McPherson Model 749 or 750 Spectrofluorometer to the new generation of HPTLC plates. Sample sizes up to 10 × 10 cm can be accommodated.*

*Several modes of operation are illustrated with a mixture of rhodamine dyes separated by HPLTC. In figure 1 direct fluorescence is shown. The developed plate was scanned from a point which includes the origin spot (0), and four developed spots. The fluorescence of each spot was monitored with fixed excitation wavelengths of 350 nm and 540 nm respectively.*

*Figure 2 illustrates fluorescence quenching. Excitation of the silica gel substrate of the plate at 265 nm results in a green background fluorescence. Absorption of 265 nm light by the various spots reduces this fluorescence, but also activates some sample fluorescence. The primary effect is a quenching of the substrate fluorescence, which is why the peak deflections are mainly negative.*

*Figure 3 demonstrates reflectance. The excitation and emission monochromators have both been set to 500 nm. Each spot absorbs some of the 500 nm light from the excitation beam, so that the reflected light of this wavelength as detected by the emission monochromator is reduced; hence, peak deflections are again negative. These two latter modes of operation are useful for compounds which absorb but do not fluoresce.*

*For information contact S.I./McPherson at 530 Main Street, Acton, Massachusetts 01720, USA. Tel.: 617 263 7733.*

**Circle No. 122 on Reader Enquiry Card**

### PNA analyser for refinery labs

An automatic gas chromatography system for hydrocarbon type analysis in petroleum fractions has been developed by Philips Analytical. The PU 4500 PNA analyser, designed by the industrial systems department at Pye Unicam Ltd, Cambridge, under licence from BP International, differs from the extremely complex systems on the market in that it is a simple, rugged, automatic method capable of producing highly repeatable results. Gas-solid chromatography with molecular sieves has become well established for light distillate analysis in the laboratories of petroleum refineries. There the efficient operation of certain processes – such as catalytic cracking or reforming – can depend on knowledge of the feedstock and product compositions in terms of hydrocarbon type (paraffins, naphthenes and aromatics) and carbon number distribution. The PU 4500 PNA analyser gives an accurate measure of the total aromatics content of a sample and of the saturated paraffins and naphthenes by carbon number up to C11. Detailed analysis of the aromatic components can easily be performed separately by conventional capillary chromatography.

*More information from Pye Unicam Ltd, York Street, Cambridge CB1 2PX, UK. Tel.: 0223 358866.*

**Circle No. 123 on Reader Enquiry Card**

### Chemical Sensors Club

A steering committee has been set up to consider the formation of a Chemical Sensors Club. The idea was a joint proposal by the UK's Laboratory of the Government Chemist and Warren Spring Laboratory. At a meeting attended by over 80 delegates representing a wide cross-section of industrial companies, research establishments and universities, the proposal to form a club to promote the development and application of chemical sensors was greeted enthusiastically.

*Organizations interested in participating, or requiring information on this rapidly developing area of research, should contact: Mr D. G. Porter, Laboratory of the Government Chemist, Cornwall House, Waterloo Road, London SE1 8XY.*

**Circle No. 124 on Reader Enquiry Card**

